# Association between omega-6 fatty acids and diabetic retinopathy risk: a systematic review and meta-analysis

**DOI:** 10.1038/s41387-026-00417-x

**Published:** 2026-06-11

**Authors:** Kai-Yang Chen, Hoi-Chun Chan, Chi-Ming Chan

**Affiliations:** 1 Department of General Medicine, Chang Gung Memorial Hospital (Linkou branch), Taoyuan, Taiwan; 2 School of Pharmacy, China Medical University, Taichung, Taiwan; 3 Department of Ophthalmology, Cardinal Tien Hospital, New Taipei City, Taiwan; 4 School of Medicine, Fu Jen Catholic University, New Taipei City, Taiwan

**Keywords:** Drug discovery, Diseases

## Abstract

**Background:**

Diabetic retinopathy (DR) is a progressive microvascular complication of diabetes and a leading cause of vision loss worldwide. Despite advances in glycemic and ophthalmologic management, additional modifiable factors influencing DR onset and progression require further exploration. Emerging evidence suggests a pivotal role of omega-6 polyunsaturated fatty acids in retinal vascular health.

**Objectives:**

To systematically synthesize and quantify associations between omega-6 fatty acids, including linoleic acid (LA), arachidonic acid (AA), and related metabolites, and the incidence, presence, progression, predictive performance, and reported adverse or non-retinal clinical outcomes of diabetic retinopathy (DR) among individuals with diabetes.

**Methods:**

A comprehensive systematic search was conducted across PubMed, Embase, Web of Science, Scopus, Cochrane Library, ScienceDirect, Wiley Online Library, EBSCO Open Research, and Google Scholar from database inception to August 15, 2025. Eligible studies included randomized trials, cohort studies, case-control studies, cross-sectional studies, and Mendelian randomization analyses that evaluated omega-6 fatty acid exposure, circulating omega-6 biomarkers, or omega-6-related metabolites in relation to DR. Quantitative synthesis was performed only for outcomes with sufficient methodological and clinical comparability. Predictive-model studies and Mendelian randomization evidence were synthesized separately. Publication bias assessments were treated as exploratory because of the small number of studies per outcome.

**Results:**

Ten studies were included (n = 1306 clinical participants plus one Mendelian randomization dataset >400,000 individuals). Higher total omega-6 levels were associated with reduced DR risk, with a random-effects odds ratio (OR) of 0.76 (95% confidence interval [CI], 0.72–0.81; I² = 53%). LA demonstrated a consistent inverse association with DR (OR = 0.78; 95% CI, 0.71–0.85; I² = 31%). In contrast, AA was associated with increased DR risk (OR = 1.15; 95% CI, 1.08–1.22; I² = 0%). Mendelian randomization supported an inverse association between genetically predicted omega-6 levels and DR risk in type 2 diabetes, with an OR of 0.75 per standard deviation (SD) increase (95% CI, 0.62–0.92). Predictive models incorporating omega-6 lipid profiles reported area under the receiver operating characteristic curve (AUC) values of 0.79–0.92; exploratory logit-transformed synthesis suggested good discrimination but should be interpreted cautiously because of methodological heterogeneity. Safety data were limited and heterogeneous, and any quantitative synthesis should be considered exploratory.

**Conclusion:**

Current evidence suggests that higher LA and related omega-6 measures may be associated with lower odds of DR, whereas higher AA and certain downstream metabolites may be associated with a higher risk. Because the evidence base remains heterogeneous in design, exposure definition, and outcome ascertainment, omega-6-related biomarkers should be regarded as promising risk-stratification candidates rather than clinically actionable markers at present.

## Introduction

Diabetic retinopathy (DR) is one of the most common and vision-threatening complications of diabetes mellitus, affecting approximately one-third of individuals with the disease [1]. It is a progressive microvascular disorder of the retina characterized by capillary leakage, ischemia, and neovascularization, which can cause irreversible vision loss [2, 3]. The increasing global prevalence of diabetes is projected to increase DR cases, with a substantial impact on public health, patient quality of life, and healthcare expenditure [4]. Current management strategies for DR, including glycemic control, systematic retinal screening, and pharmacologic or procedural ophthalmic interventions, have reduced vision-threatening complications but have not eliminated the overall disease burden [5]. Recent systematic reviews, including our prior work on fibrates and comparative treatment strategies for DR, suggest that metabolic and vascular modulation can influence retinal outcomes, yet substantial residual risk persists [6, 7]. These observations support continued investigation of additional modifiable factors, including polyunsaturated fatty acids (PUFAs), that may influence retinal microvascular vulnerability and disease progression [8].

PUFAs are essential components of cell membrane structure, energy metabolism, and the regulation of inflammatory pathways [9]. Omega-6 fatty acids include linoleic acid (LA), which can be metabolized into bioactive derivatives such as arachidonic acid (AA) and dihomo-γ-linolenic acid (DGLA) [10]. Omega-6 fatty acids exhibit dual mechanisms, including both pro-inflammatory and anti-inflammatory effects [11]. This makes it challenging to characterize their role in the pathophysiology of microvascular complications such as DR.

Omega-6 fatty acids are involved in vascular homeostasis, platelet aggregation, and inflammatory signaling, which influence retinal microvascular characteristics [12]. In addition, dietary patterns and circulating fatty acid profiles have been increasingly linked to diabetes outcomes, highlighting the significance of the balance of fatty acid intake in DR risk [13]. Evidence suggests a protective association between higher levels of LA or DGLA and a reduced incidence of DR [10]. In contrast, some studies link elevated AA metabolites to disease progression [14]. These findings suggest that omega-6 fatty acids and their metabolites may represent modifiable biomarkers or candidate therapeutic pathways in DR.

Additionally, AA-derived metabolites, such as prostaglandins and leukotrienes, can promote inflammation, oxidative stress, and endothelial dysfunction, contributing to retinal vascular injury [15, 16]. These mechanisms are consistent with broader pathogenic pathways in DR, in which metabolic stressors such as methylglyoxal, mitochondrial dysfunction, and dysregulated autophagy drive retinal pigment epithelial and microvascular damage—processes we and others have previously characterized in experimental models [17–19]. 12-hydroxyeicosatetraenoic acid (12-HETE) has been implicated in promoting leukocyte adhesion and vascular inflammation in the retina [20]. In contrast, omega-6 derivatives such as DGLA may induce anti-inflammatory prostaglandins, while dietary LA enrichment may improve lipid metabolism and reduce microvascular damage in diabetic patients [21]. These dual mechanisms suggest that the impact of omega-6 fatty acids on DR may depend on specific metabolites and dietary composition.

Despite growing research interest, the current evidence base regarding omega-6 fatty acids and DR remains inconclusive. Our group previously demonstrated, through a systematic review and meta-analysis, that higher omega-3 fatty acid intake is associated with a reduced risk of DR, underscoring the importance of PUFAs in retinal microvascular protection [22]. However, most prior work has focused on omega-3 pathways, whereas the role of omega-6 fatty acids—particularly the balance between protective species such as LA and potentially harmful metabolites like AA—has not been comprehensively quantified. This systematic review aimed to synthesize the available evidence on the association between omega-6 fatty acids and DR across multiple evidence domains, including dietary exposure, circulating biomarkers, omega-6-derived metabolites, predictive lipid signatures, and genetically predicted omega-6 levels. The primary objectives were to evaluate associations with DR incidence, presence, progression, and severity, and to summarize the available evidence on predictive performance and reported adverse outcomes where applicable.

## Research purpose

To critically appraise and synthesize the evidence on the association between omega-6 fatty acid-related exposures and DR in individuals with diabetes.

## Methodology

### Protocol and registration

The protocol for this project was registered in the International Prospective Register of Systematic Reviews (PROSPERO) (CRD420251134144). The study was prepared, conducted, and reported according to the Preferred Reporting Items for Systematic Reviews and Meta-Analyses (PRISMA) [23].

### Data sources

One reviewer (K-YC) conducted a comprehensive search of studies published in the English-language literature investigating associations between omega-6 fatty acid-related exposures and DR onset, presence, or progression. The electronic database search was performed using PubMed, Google Scholar, ScienceDirect, Scopus, Embase, EBSCO Open Research, Wiley Online Library, Web of Science, and Cochrane Library to retrieve peer-reviewed original research articles published from inception to August 15, 2025.

### Search strategy

A comprehensive search strategy was developed to identify studies evaluating omega-6 fatty acids and diabetic retinopathy. Preliminary scoping searches were performed in PubMed and Google Scholar to identify relevant controlled vocabulary terms and free-text keywords (Table 1). The final database-specific strategies combined terms for diabetic retinopathy with polyunsaturated fatty acid-related terms, including omega-6 fatty acids, linoleic acid, arachidonic acid, dihomo-γ-linolenic acid, and related fatty acid terms. Because omega-6 and omega-3 fatty acids are frequently reported together in nutritional and lipidomic studies, broader polyunsaturated fatty acid terms were retained in several search strings to maximize sensitivity. Search strings were adapted for each database according to indexing structure and syntax.

**Table 1:** Search strategies for omega-6 fatty acids and DR.

Database	Search strategy (Boolean Operators, MeSH/Keywords)
**PubMed**	(“Diabetic Retinopathy”[MeSH] OR “diabetic retinopathy”[tiab] OR “retinal microangiopathy”[tiab] OR “retinal microvascular”[tiab]) AND (“Fatty Acids, Omega-3”[MeSH] OR “Fatty Acids, Omega-6”[MeSH] OR “omega-3”[tiab] OR “omega-6”[tiab] OR “n-3 PUFA”[tiab] OR “n-6 PUFA”[tiab] OR “linoleic acid”[tiab] OR “arachidonic acid”[tiab] OR “dihomo-gamma-linolenic acid”[tiab] OR “eicosapentaenoic acid”[tiab] OR “docosahexaenoic acid”[tiab]) AND (“Diabetes Mellitus”[MeSH] OR “diabetes mellitus”[tiab] OR “type 1 diabetes”[tiab] OR “type 2 diabetes”[tiab] OR “T1DM”[tiab] OR “T2DM”[tiab])
**EBSCO Open Research**	(“diabetic retinopathy” OR “retinal microangiopathy” OR “retinal vascular complication”) AND (“omega-3 fatty acids” OR “omega-6 fatty acids” OR “n-3 PUFA” OR “n-6 PUFA” OR “linoleic acid” OR “arachidonic acid” OR “dihomo-gamma-linolenic acid” OR “EPA” OR “DHA”) AND (diabetes OR “type 1 diabetes” OR “type 2 diabetes”)
**ScienceDirect**	(“diabetic retinopathy”) AND (“omega-3” OR “omega-6”) AND (“diabetes” OR “type 1 diabetes” OR “type 2 diabetes”) AND (“predictive model”)
**Wiley Online Library**	(“diabetic retinopathy” OR “retinal microangiopathy” OR “retinal vascular damage”) AND (“omega-3” OR “omega-6” OR “n-3 PUFA” OR “n-6 PUFA” OR “linoleic acid” OR “arachidonic acid” OR “EPA” OR “DHA” OR “DGLA”) AND (diabetes OR “type 1 diabetes” OR “type 2 diabetes”)
**Google Scholar**	“diabetic retinopathy” AND (“omega-3” OR “omega-6” OR “PUFA” OR “polyunsaturated fatty acid” OR “linoleic acid” OR “arachidonic acid” OR “EPA” OR “DHA”) AND (“diabetes” OR “type 1 diabetes” OR “type 2 diabetes”)
**Cochrane Library**	(diabetic retinopathy OR retinal microangiopathy OR retinal vascular complication) AND (omega-3 fatty acids OR omega-6 fatty acids OR n-3 PUFA OR n-6 PUFA OR linoleic acid OR arachidonic acid OR EPA OR DHA OR DGLA) AND (diabetes OR type 1 diabetes OR type 2 diabetes)
**EMBASE (Ovid)**	(‘diabetic retinopathy’/exp OR ‘retinal microangiopathy’:ab,ti OR ‘retinal vascular complication’:ab,ti) AND (‘omega 3 fatty acid’/exp OR ‘omega 6 fatty acid’/exp OR ‘n 3 PUFA’:ab,ti OR ‘n 6 PUFA’:ab,ti OR ‘linoleic acid’:ab,ti OR ‘arachidonic acid’:ab,ti OR ‘DGLA’:ab,ti OR ‘EPA’:ab,ti OR ‘DHA’:ab,ti) AND (‘diabetes mellitus’/exp OR ‘type 1 diabetes’:ab,ti OR ‘type 2 diabetes’:ab,ti)
**Web of Science**	“diabetic retinopathy” OR “retinal microangiopathy” OR “retinal vascular complication” AND (“omega-3” OR “omega-6” OR “n-3 PUFA” OR “n-6 PUFA” OR “linoleic acid” OR “arachidonic acid” OR “EPA” OR “DHA” OR “DGLA”) AND (“diabetes” OR “type 1 diabetes” OR “type 2 diabetes”) AND (“predictive Models” OR “Models”)
**SCOPUS**	(“diabetic retinopathy”) AND (“omega-3” OR “omega-6”) AND (“diabetes” OR “type 1 diabetes” OR “type 2 diabetes”) AND (“predictive model”)

Database-specific Boolean operators, Medical Subject Headings (MeSH) terms, and keyword combinations used to identify eligible studies.

*DR* diabetic retinopathy, *PUFA* polyunsaturated fatty acid, *EPA* eicosapentaenoic acid, *DHA* docosahexaenoic acid, *DGLA* dihomo-γ-linolenic acid, *T1DM* type 1 diabetes mellitus, *T2DM* type 2 diabetes mellitus.

### Study selection and screening

The study selection and screening process was conducted using Zotero reference management software version 6.0.36 for efficient and transparent reference management. All retrieved records from the database searches were imported into Zotero, where duplicates were automatically identified by comparing the metadata of the records and manually merged. Additionally, records flagged as retracted during screening were manually removed.

A two-stage screening process, including title and abstract screening and full-text screening, was then conducted. In the title and abstract screening step, two independent research authors (K-YC and H-CC) screened the studies against predefined inclusion and exclusion criteria to determine their potential relevance. Discrepancies were resolved through discussion until consensus was reached or by involving a third independent research author (C-MC), whenever necessary. Studies deemed potentially eligible were retrieved for the full-text screening step, where the full articles were assessed against the same eligibility criteria. Reasons for exclusion at the full-text stage were systematically documented using a PRISMA flow diagram.

### Eligibility criteria

This study included original peer-reviewed journal research articles that investigated associations between omega-6 fatty acid-related exposures and the onset, presence, progression, severity, or prediction of DR.

### Inclusion criteria

A modified PICOS framework was used to define the inclusion criteria as follows [24].

Population (P): Human participants with diabetes, with or without DR; studies including healthy controls were also eligible when used for biomarker comparison.

Intervention (I): Omega-6 fatty acid-related exposures, including dietary intake, dietary supplementation, circulating omega-6 fatty acids, erythrocyte membrane omega-6 composition, omega-6-derived metabolites, or genetically predicted omega-6 levels.

Comparison (C): Diabetic participants without retinopathy, diabetic participants with a different stage of retinopathy, healthy controls, or lower versus higher omega-6 exposure categories.

Outcomes (O): Incident DR, prevalent DR, retinopathy progression, retinopathy severity, or predictive performance for DR.

Study Design (S): Randomized controlled trials, cohort studies, case-control studies, cross-sectional studies, and Mendelian randomization analyses. Additional eligibility rule: studies assessing only non-specific total free fatty acids or broad metabolomic profiles without extractable omega-6-specific measures were excluded from quantitative synthesis and considered for qualitative discussion only if directly relevant.

### Exclusion criteria

Non-human studies, including animal models or in vitro research, were excluded. In addition, studies were excluded if they were review articles, editorials, commentaries, conference abstracts, book chapters, secondary analyses, or opinion pieces. Moreover, non-English publications and articles without access to the full texts were excluded.

### Risk of bias assessment

Two independent research authors (K-YC and H-CC) evaluated the methodological quality and internal validity of the eligible studies. Risk of bias was assessed according to study design. Randomized dietary intervention evidence was assessed using the Cochrane Risk of Bias 2 (RoB 2) tool, observational studies were assessed using the Newcastle-Ottawa Scale (NOS), and Mendelian randomization evidence was interpreted separately because it addresses a genetic causal estimand rather than a conventional clinical trial exposure. The Newcastle-Ottawa Scale evaluated selection, comparability, and outcome or exposure ascertainment domains, depending on study design [25]. Because Table 2 presents a simplified domain-level display, comparability and confounding concerns were additionally considered in the narrative interpretation. The RoB 2 tool assessed bias arising from the randomization process, deviations from the intended intervention, missing outcome data, measurement of the outcome, and selection of the reported result [26]. Discrepancies were resolved through discussion until consensus was reached or by involving a third independent research author (C-MC), whenever necessary.

### Data extraction

For each study meeting the inclusion criteria, two review authors (K-YC and H-CC) independently extracted the data from each article. One review author checked the extracted data for consistency and compiled it into a single final form. In the event of inconsistencies, the review authors discussed the article to reconcile any conflicts until a consensus was achieved. A third senior author (C-MC) was consulted to facilitate the reconciliation process whenever necessary.

The following data were extracted: author (year), country, study design, sample size, population characteristics, omega-6 intervention, comparator/control, primary outcome(s), assessment methods, and findings.

### Handling missing data

During data extraction, in case critical information was not reported or was unclear, attempts were made to contact the corresponding authors via email to request the missing information. If no response was received after two attempts, the available data were used as reported, and the potential impact of the missing information was noted.

When quantitative data necessary for meta-analysis were missing, alternative strategies such as calculating estimates from available statistics or extracting values from figures were considered, following Cochrane guidelines. However, studies with missing outcome data that could not be reasonably addressed were included in the qualitative synthesis but excluded from the quantitative synthesis. The extent and potential impact of missing data were reported and discussed in the review’s limitations section.

## Data analysis

### Qualitative synthesis

A qualitative synthesis was conducted to summarize the key characteristics and findings of the included studies. In addition, qualitative findings were synthesized through thematic analysis, identifying recurring concepts, patterns, and themes related to the use and effects of omega-6 fatty acids in patients with DR [27]. The extracted data were systematically coded and grouped into the following themes: DR prediction and management, and safety and adverse events related to omega-6 supplementation.

### Meta-analysis

A research author (K-YC) conducted a meta-analysis using Comprehensive Meta-Analysis (CMA, Version 3.0) software, where sufficient homogeneity in population, interventions, comparisons, and outcomes was established. Effect measures were analyzed as odds ratios (ORs), proportions, rates, or logit-transformed estimates, depending on the outcome and available data, with corresponding 95% confidence intervals (CIs). Both fixed-effects and random-effects models (DerSimonian–Laird method) were applied to account for within- and between-study variability. Cochran’s Q-statistic and I² index were used to assess heterogeneity, with I² values of <25%, 25–75%, and >75% representing low, moderate, and high heterogeneity, respectively. When high heterogeneity was observed, extracted data and coding decisions were rechecked before final analyses were reported. Statistical significance was determined at *p* < 0.05 (two-tailed).

Publication bias was evaluated using Begg and Mazumdar’s rank correlation test, Egger’s regression test, and Duval and Tweedie’s trim-and-fill method, complemented by funnel plot visualization to assess asymmetry. For predictive-model outcomes, individual area under the receiver operating characteristic curve (AUC), sensitivity, and specificity values were summarized, and exploratory logit-transformed estimates were reported where appropriate. Where possible, subgroup estimates were compared across biomarker types (LA, AA, and total omega-6 PUFAs) and outcome domains (incidence, progression, predictive accuracy, and safety). All statistical procedures followed PRISMA 2020 and MOOSE reporting standards to ensure methodological transparency and reproducibility.

## Results

### Study selection and screening

**Fig. 1:**
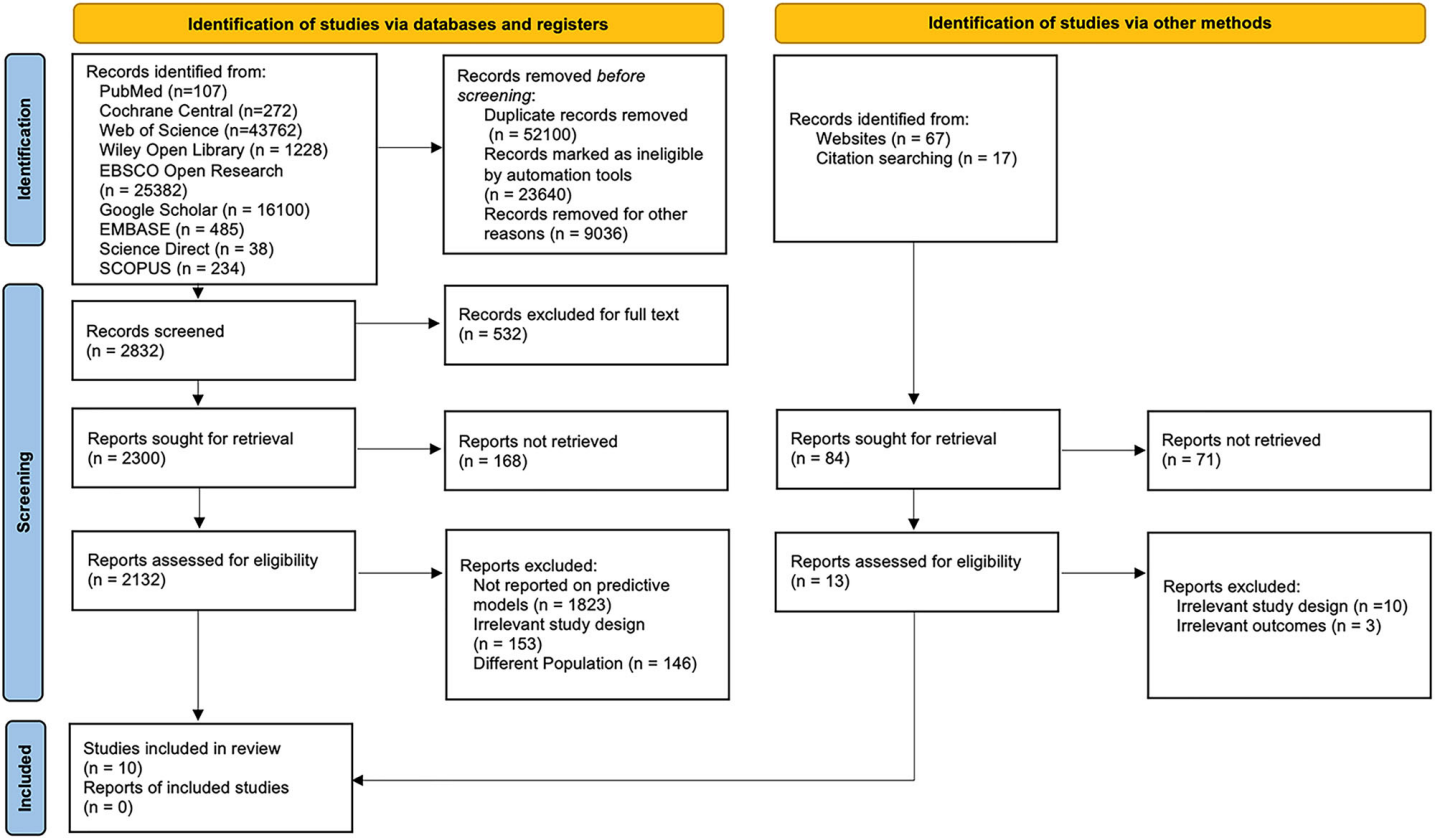
Preferred Reporting Items for Systematic Reviews and Meta-Analyses (PRISMA) 2020 flow diagram of study selection.

The PRISMA flow diagram summarizes the identification, screening, eligibility assessment, and inclusion of studies. Ten studies met the eligibility criteria and were included in the final review (Fig. 1).

### Risk of bias assessment

**Fig. 2:**
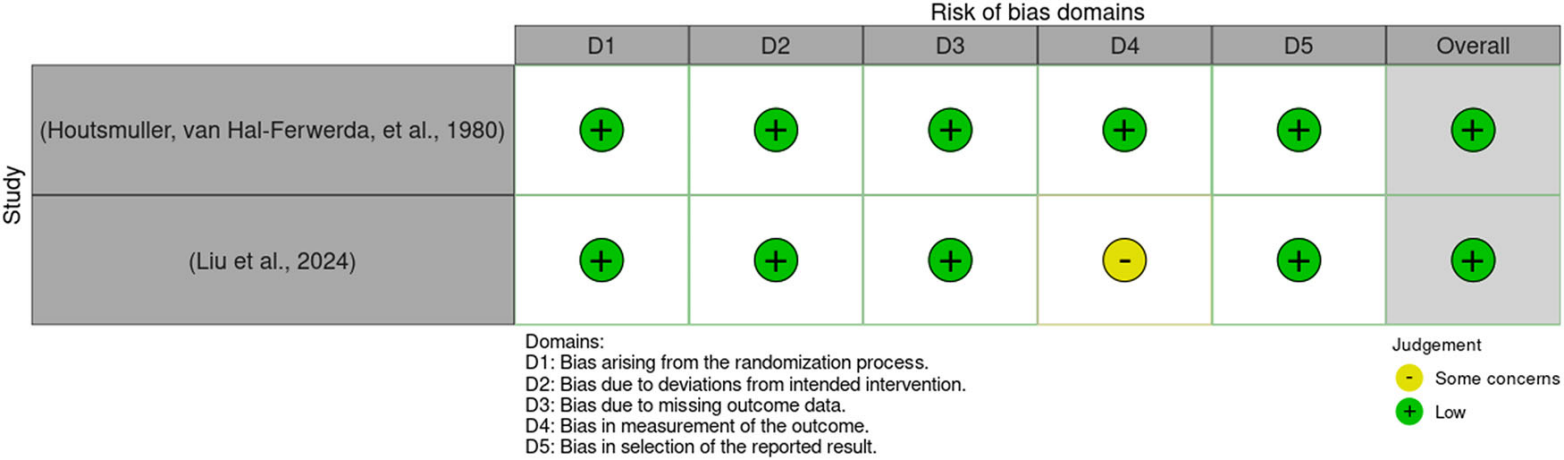
Risk of Bias 2 (RoB 2) traffic light plot for randomized dietary intervention studies.

**Fig. 3:**
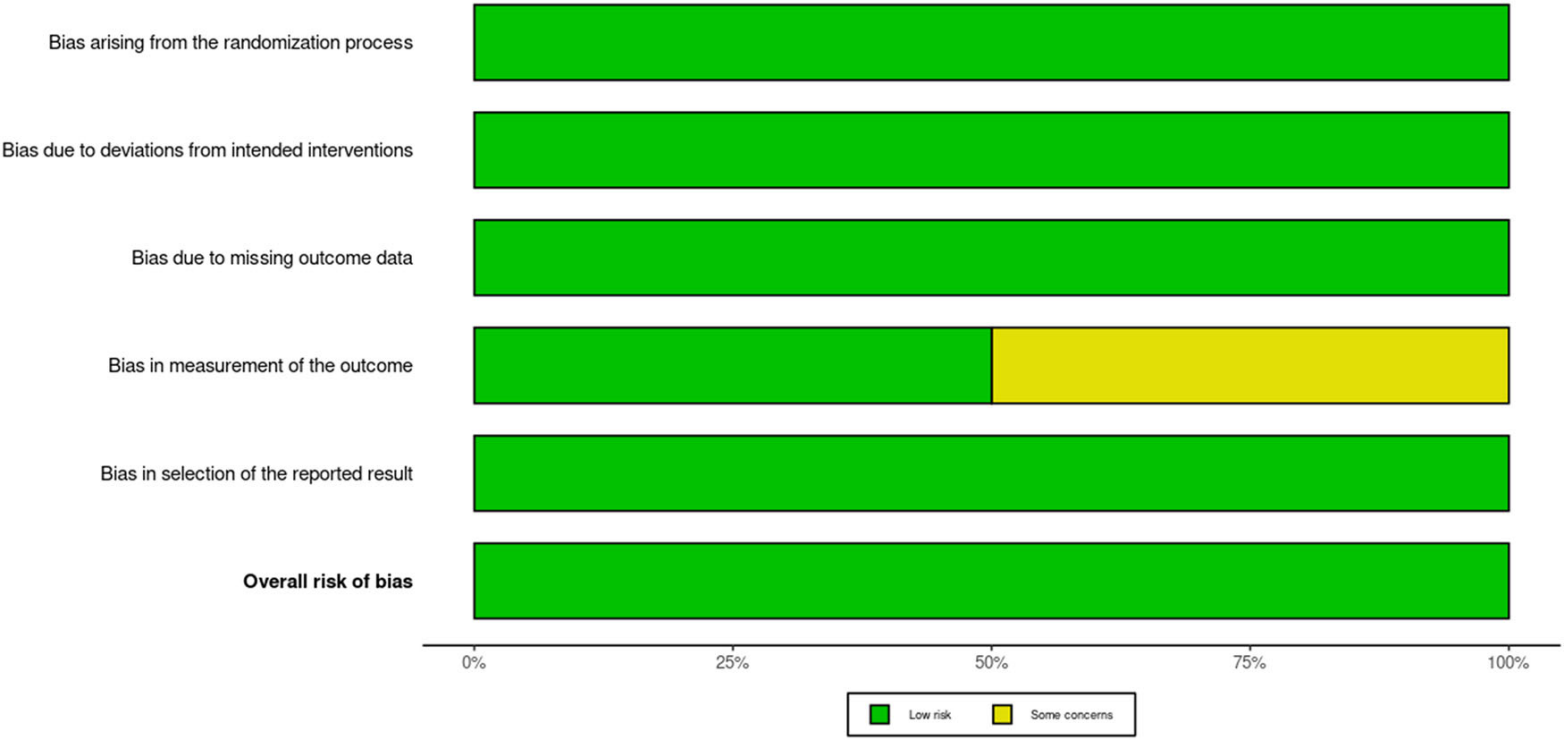
Risk of Bias 2 (RoB 2) summary plot for randomized dietary intervention studies.

The included studies generally showed acceptable methodological quality across the assessed domains, although risk-of-bias interpretation was limited by heterogeneity in study design, as shown in Figs. 2 and 3 and Table 2.

**Table 2:** Simplified Newcastle-Ottawa Scale domain-level assessment of observational studies.

Study	Selection		Outcome	
	Representativeness of the cohort	Ascertainment of exposure	Assessment of outcomes	Follow-up
(Chen et al. 2024) [28]	☆	☆	☆	☆
(Feng et al. 2023) [29]	☆	☆	☆	☆
(Houtsmuller, Zahn, et al. 1980) [31]	☆	☆	☆	☆
(Karagöz et al. 2021) [32]	☆	☆	☆	☆
(Koehrer et al. 2014) [33]	☆	☆	☆	☆
(Li et al. 2020) [34]	☆	☆	☆	☆
(Okamura et al. 2020) [36]	☆	☆	☆	☆
(Zhao, et al. 2021) [37]	☆	☆	☆	☆

Simplified quality evaluation of cohort and case-control studies across displayed selection and outcome/exposure domains. Comparability and confounding concerns were considered in the narrative interpretation. *NOS* Newcastle-Ottawa Scale.

### Data selection and extraction

#### Overview of study characteristics

This review included 10 studies spanning multiple evidence domains and populations from China, the Netherlands, Turkey, France, Japan, the United Kingdom, and Finland. The clinically characterized studies together enrolled 1306 participants, while one Mendelian randomization analysis used large-scale genetic summary data from population cohorts. The included evidence comprised dietary intervention studies, cohort studies, case-control studies, cross-sectional biomarker studies, and one Mendelian randomization study. Because these designs address different estimands and differ substantially in exposure ascertainment and susceptibility to bias, they were interpreted according to study design rather than treated as a single homogeneous body of evidence. Sample sizes in clinical studies ranged from 74 to 240 participants. Some studies measured circulating omega-6 fatty acids, including AA, LA, and DGLA, using advanced techniques such as liquid chromatography-tandem mass spectrometry and ultra-performance liquid chromatography-mass spectrometry, while others involved dietary interventions with omega-6-rich diets. Assessment methods included fundus examinations, fluorescein angiography, and standardized DR classification systems (Table 3).

**Table 3:** Characteristics of included studies.

Author (Year)	Country	Study design	Sample size	Population characteristics	Omega-6 intervention	Comparator / Control	Primary Outcome(s)	Assessment Methods	Findings
(Chen et al. 2024) [28]	China	Cohort study	74	Adults with T2DM, no DR at baseline	Serum 12-HETE (omega-6 metabolite of AA)	Low vs. High serum 12-HETE levels	Incidence of DR (new-onset DR)	UPLC-MS (12-HETE); logistic regression; qPCR; ELISA; WB; leukocyte adhesion assay	Elevated 12-HETE levels are linked to increased DR incidence and appear to promote retinal endothelial inflammation through the GPR31/p38 MAPK pathway
(Feng et al. 2023) [29]	China	Cohort study	240 (Training: HC = 82, DR = 58, NDR = 60; Validation: DR = 20, NDR = 20)	Adults with T2DM with and without DR; healthy controls	No supplementation	Healthy controls, T2DM without DR (NDR) vs T2DM with DR	Ability of serum omega-6 fatty acids and bile acids to predict DR in T2DM patients	LC-MS/MS for UFAs and BAs; fundus exams for DR diagnosis; OPLS-DA, ROC, IDI, NRI, multivariate logistic regression, Spearman correlations	AA, alongside TLCA and TUDCA, emerged as independent predictors of DR
(Houtsmuller, van Hal-Ferwerda, et al. 1980) [30]	Netherlands	RCT	102	Adults with diabetes	Diet with 40% fat, ⅓ of which LA (4× higher than control)	Diet with 35% saturated fat, low LA (omega-6 poor)	Incidence/progression of DR	Retinopathy incidence, GTTs, insulin levels, cardiac events, lipid profiles	LA supplementation was associated with reduced retinopathy progression and improved cardiac outcomes, particularly in females, without influencing platelet aggregation.
(Houtsmuller, Zahn, et al. 1980) [31]	Netherlands	Cohort study	96	Adults with newly diagnosed type 2 diabetes	Diet II: 45% carbs, 40% fats (1/3 LA), 15% protein—20 g LA/1000 kcal	Diet I: 50% carbs, 35% saturated fat, 15% protein—5 g LA/1000 kcal	Progression of DR (fluorescein-based staging 0–3)	Fluorescein angiography; GTT + insulin; retinopathy stages 0–3 recorded over time	A long-term omega-6-rich diet was associated with significant protection against DR progression, especially among those with poor glycemic control
(Karagöz et al. 2021) [32]	Turkey	Retrospective cohort study	157	Patients with type 2 diabetes mellitus (T2DM), divided into 3 groups by DR severity	AA-based platelet reactivity test (ASPI)	Groups: No DR (n = 49), NPDR (n = 58), PDR (n = 50)	Association between ASPI/ADP platelet reactivity and DR severity	Multiplate whole blood aggregometry (ASPI and ADP tests), full ophthalmological examination and DR staging based on the modified Airlie-House classification	Increased platelet reactivity and elevated HbA1c were associated with greater DR severity
(Koehrer et al. 2014) [33]	France	Case-control study	102	Adults: controls and mostly type 2 diabetic patients with/without varying stages of DR	No supplementation	Control group vs DR patients (mild to proliferative)	Erythrocyte levels of omega-6 (AA) and omega-3 (DHA) phospholipids	Gas chromatography for fatty acids, LC-ESI-MS/MS for phospholipid structure, ETDRS classification for DR severity, Kruskal-Wallis and post-hoc Dunn’s tests	Reduced levels of AA and DHA in DR patients were linked to the loss of specific phospholipid species
(Li et al. 2020) [34]	China	Case-control study	138 (69 DR cases, 69 DM controls)	Adults with Type 2 Diabetes	No supplementation	Type 2 diabetes patients without DR (matched by age, sex, BMI, HbA1c)	Association between serum n-6 PUFA levels and DR risk	UPLC-ESI-MS/MS for PUFA profiling; ophthalmic examination; logistic and principal component regression analysis	Elevated serum levels of omega-6 PUFAs, particularly LA, gamma-linolenic acid, and dihomo-gamma-linolenic acid, were strongly associated with reduced presence and severity of DR
(Liu et al. 2024) [35]	UK & Finland	Mendelian randomization study	Exposure GWAS: ~110,000 (UK Biobank); Outcome GWAS: ~310,000–313,000 (FinnGen)	Individuals with Type 1 or Type 2 Diabetes	Genetically predicted omega-6 fatty acid levels	Genetically predicted omega-6 fatty acid levels	DR in type 2 diabetes	MR using inverse-variance weighted (IVW), weighted median, MR-Egger regression, multivariable MR (MVMR)	Genetic evidence suggests a potential protective role of higher omega-6 levels against DR and nephropathy in type 2 diabetes, with no observed effect in type 1 diabetes and no safety data reported.
(Okamura et al. 2020) [36]	Japan	Cohort study	190	Japanese patients with T2DM	Circulating omega-6 PUFA: DGLA measured via GC-MS	Patients with and without DR (NDR vs SDR/PDR)	Presence and severity of DR (NDR, SDR, PDR)	DR diagnosis Davis classification, Fatty acids measured via GC-MS, Diet assessed via BDHQ, Statistical analysis	Lower levels of DGLA were observed in patients with DR, and its inverse association with the condition indicates a potential protective role.
(Zhao, et al. 2021) [37]	China	Case-control study	207 total (69 DR, 69 DM, 69 healthy)	Adults with type 2 diabetes (with and without DR)	Measured serum omega-6 PUFAs (LA, GLA, DGLA, AA); calculated omega-6/omega-3 ratio (PUFAR)	T2D patients with DR vs without DR vs healthy controls	Association between serum omega-6/omega-3 ratio (PUFAR) and presence of DR	Serum PUFA quantified via UPLC-MS/MS; DR diagnosed via standardized clinical ophthalmologic exams	Higher serum omega-6 levels, particularly LA, and an elevated PUFAR were independently linked to reduced likelihood of DR

Summary of study design, population, omega-6 exposure, comparators, outcomes, and main findings. *T2DM* type 2 diabetes mellitus, *T2D* type 2 diabetes, *DR* diabetic retinopathy, *12-HETE* 12-hydroxyeicosatetraenoic acid, *AA* arachidonic acid, *GPR31* G protein-coupled receptor 31, *MAPK* mitogen-activated protein kinase, *HC* healthy control, *NDR* no diabetic retinopathy, *LC-MS/MS* liquid chromatography-tandem mass spectrometry, *UFA* unsaturated fatty acid, *BA* bile acid, *OPLS-DA* orthogonal partial least squares discriminant analysis, *ROC* receiver operating characteristic, *IDI* integrated discrimination improvement, *NRI* net reclassification improvement, *TLCA* taurolithocholic acid, *TUDCA* tauroursodeoxycholic acid, *RCT* randomized controlled trial, *LA* linoleic acid, *GTT* glucose tolerance test, *ASPI* arachidonic acid-induced platelet aggregation test, *ADP* adenosine diphosphate, *NPDR* non-proliferative diabetic retinopathy, *PDR* proliferative diabetic retinopathy, *HbA1c* glycated hemoglobin, *DHA* docosahexaenoic acid, *LC-ESI-MS/MS* liquid chromatography-electrospray ionization-tandem mass spectrometry, *ETDRS* Early Treatment Diabetic Retinopathy Study, *BMI* body mass index, *UPLC-ESI-MS/MS* ultra-performance liquid chromatography-electrospray ionization-tandem mass spectrometry, *PUFA* polyunsaturated fatty acid, *GLA* γ-linolenic acid, *DGLA* dihomo-γ-linolenic acid, *GWAS* genome-wide association study, *IVW* inverse-variance weighted, *MVMR* multivariable Mendelian randomization, *GC-MS* gas chromatography-mass spectrometry, *BDHQ* brief-type self-administered diet history questionnaire, *SDR* simple diabetic retinopathy, *PUFAR* omega-6/omega-3 polyunsaturated fatty acid ratio.

### Systematic review results

#### DR prediction and management

Studies reported metabolite-specific associations between omega-6 fatty acid-related exposures and DR, with generally inverse associations for LA or DGLA and positive associations for AA or 12-HETE-related pathways. 12-HETE demonstrated an optimal predictive threshold of 2.235 ng/mL with an area under the curve of 0.836 (95% CI: 0.764-0.909), 69.6% sensitivity, and 97.3% specificity for DR onset prediction. There were significant differences in disease incidence, with only 2.99% of patients in the Low 12-HETE group developing DR, compared to 42.86% in the High 12-HETE group [28]. A model using AA, TLCA, and TUDCA achieved high predictive modeling accuracy, with an AUC of 0.785 in the training cohort (95% CI: 0.704-0.866) and improved to 0.918 in the validation cohort [29]. A LA-rich diet significantly reduced DR progression; 62% of males and 55% of females in the low-linoleic group developed DR, compared to only 27% of males (*p* < 0.001) and 32% of females (*p* < 0.025) in the high-linoleic group [30].

LA-rich dietary exposure appeared more strongly protective in high-risk participants with persistently elevated blood glucose levels. Among patients with persistently elevated blood sugar, 7 out of 8 males and all 5 females on the LA-poor diet exhibited disease progression, while none of the 11 patients on the LA-rich diet showed any worsening of their retinopathy [31]. Increased ASPI (OR: 1.044; 95% CI: 1.021–1.09; *p* < 0.001) and ADP values (OR: 1.033; 95% CI: 1.010–1.10; *p* = 0.003) were significantly associated with higher DR stage, with binary logistic regression indicating that ASPI (OR: 1.061; 95% CI: 1.031–1.10; *p* < 0.001) and ADP (OR: 1.03; 95% CI: 1.01–1.06; *p* = 0.045) predicted the presence of DR [32]. Circulating omega-6 levels were lower in DR groups than in controls, with nonmonotonic variation across retinopathy severity strata: 29.92% in controls, 24.94% in mild retinopathy, 21.40% in moderate retinopathy, and 24.86% in severe retinopathy cases [33].

In addition, total n-6 PUFAs were significantly associated with a 71% reduction in DR risk (aOR 0.29; 95% CI 0.13–0.64) and showed excellent discrimination with an AUC of 0.88 (95% CI 0.78–0.99) in the testing set [34].

Mendelian randomization evidence showed a statistically significant reduction in DR risk in type 2 diabetes associated with a one standard deviation increase in genetically predicted omega-6 levels (OR, 0.75; 95% CI, 0.62–0.92; *p* = 0.005) [35]. In the Okamura cohort, 85.8% of participants had no retinopathy, 6.8% had simple DR, and 7.4% had proliferative DR; higher log-transformed DGLA levels were associated with lower odds of DR. In addition, each one-unit increase in log-transformed DGLA levels was significantly associated with reduced odds of DR (unadjusted OR 0.76; 95% CI: 0.62–0.96, *p* = 0.020) [36]. In another study, total omega-6 PUFAs were inversely associated with DR, with each one standard deviation increase linked to a 50% reduction in odds (OR, 0.50; 95% CI, 0.33–0.75). The AUC for the PUFA ratio in detecting DR was 0.83 in the training set and 0.79 in the testing set, with sensitivity ranging from 71.4% to 75.0%, specificity ranging from 77.1% to 80.9%, positive predictive values ranging from 76.6% to 78.9%, and negative predictive values ranging from 73.9% to 75.5%, indicating promising predictive performance [37].

#### Safety and adverse events related to omega-6 supplementation

The exploratory synthesis of reported adverse or non-retinal clinical outcomes is shown in Fig. 8. Because the included studies differed in exposure type, outcome definition, follow-up duration, and adverse-event ascertainment, these data should be interpreted descriptively rather than as definitive evidence of safety. The available studies did not identify a consistent signal suggesting increased adverse outcomes with higher LA exposure, but the limited number and heterogeneity of studies preclude firm conclusions regarding safety.

### Meta-analysis results

#### DR occurrence or presence in relation to omega-6 fatty acid measures

**Fig. 4:**
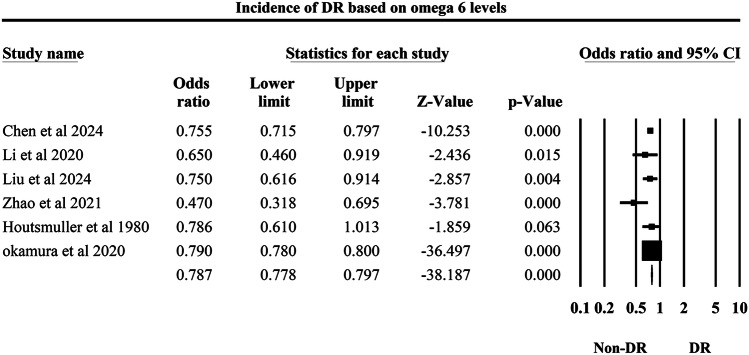
Forest plot of omega-6 fatty acids and diabetic retinopathy (DR) occurrence or presence. CI, confidence interval.

The pooled analysis of six studies, shown in Fig. 4, demonstrated an inverse association between omega-6 fatty acid measures and DR occurrence or presence among diabetic patients. The combined fixed-effects estimate yielded an OR of 0.79 (95% CI: 0.78–0.80; *p* < 0.001), while the random-effects model produced a comparable result (OR = 0.76; 95% CI: 0.72–0.81; *p* < 0.001). An OR below 1 indicates that higher circulating or dietary omega-6 fatty acids are associated with lower odds of DR occurrence or presence compared with lower omega-6 exposure. Among individual studies, Chen et al. (2024) and Zhao et al. (2021) contributed the largest weights to the pooled estimate due to their robust sample sizes and narrow CIs, while Li et al. (2020) and Liu et al. (2024) provided consistent directional effects. Heterogeneity analysis revealed moderate-to-low variability (Q = 10.71, df = 5, *p* = 0.057; I² = 53.3%), indicating acceptable consistency across studies and supporting the reliability of the pooled estimate. Clinically, these findings suggest that maintaining adequate omega-6 exposure, particularly LA and its derivatives, may be associated with preservation of retinal microvascular integrity and lower odds of DR in diabetic populations. The observed heterogeneity supports cautious interpretation of omega-6 fatty acid measures as potential protective nutritional biomarkers for diabetic microvascular complications.

#### DR occurrence or presence in relation to LA levels

**Fig. 5:**
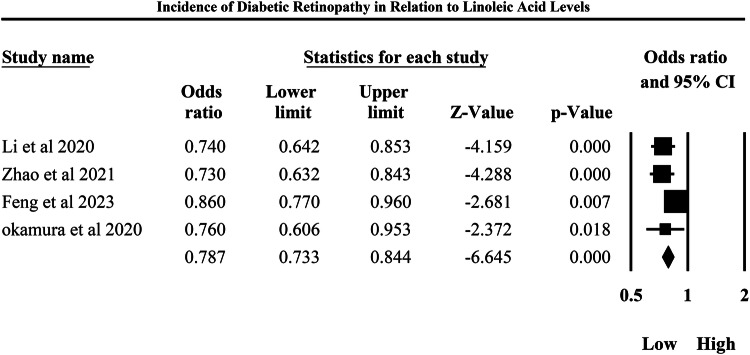
Forest plot of linoleic acid (LA) and diabetic retinopathy (DR) occurrence or presence. CI, confidence interval.

The pooled analysis of four studies, shown in Fig. 5, demonstrated a consistent and statistically significant inverse association between LA levels and DR occurrence or presence among diabetic patients. The fixed-effects model produced an overall OR of 0.79 (95% CI: 0.73–0.84; *p* < 0.001), while the random-effects model yielded a comparable estimate (OR = 0.78; 95% CI: 0.71–0.85; *p* < 0.001). An OR below 1 indicates that higher LA levels are associated with approximately 21–22% lower odds of DR compared with lower LA exposure. Among the included studies, Li et al. (2020) and Zhao et al. (2021) contributed the largest weights to the pooled effect due to larger sample sizes and narrow CIs, while Feng et al. (2023) and Okamura et al. (2020) demonstrated directionally consistent results. Heterogeneity was low (Q = 4.35, df = 3, *p* = 0.23; I² = 31.0%), suggesting strong agreement among studies and supporting the robustness of the pooled estimate. Clinically, these findings highlight LA as a candidate biomarker associated with lower odds of DR in diabetic patients. These findings support further evaluation of LA-related dietary and biomarker pathways in relation to retinal microvascular outcomes.

#### DR occurrence or presence in relation to AA levels

**Fig. 6:**
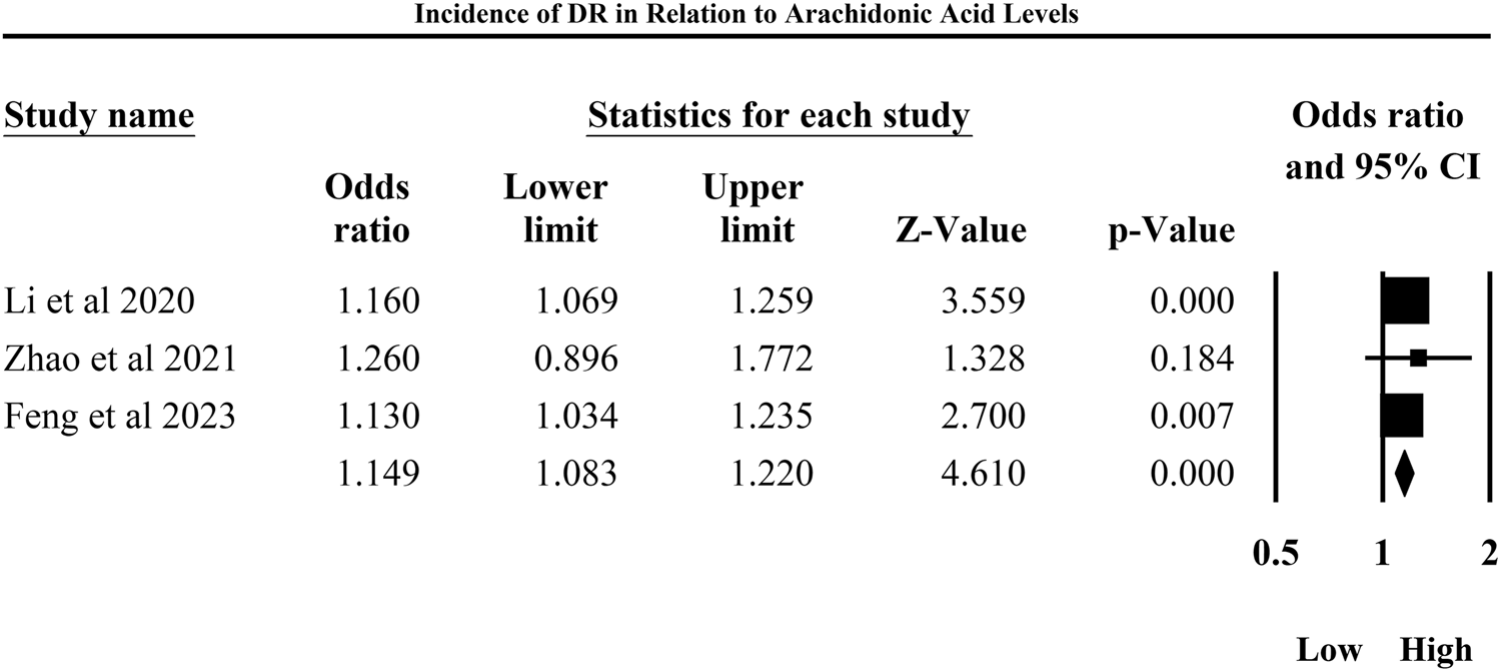
Forest plot of arachidonic acid (AA) and diabetic retinopathy (DR) occurrence or presence. CI, confidence interval.

The pooled analysis of three studies, shown in Fig. 6, revealed a statistically significant positive association between AA levels and DR occurrence or presence among diabetic patients. The combined fixed-effects estimate demonstrated an OR of 1.15 (95% CI, 1.08–1.22; *p* < 0.001), indicating that higher circulating AA levels are associated with 15% higher odds of DR compared with lower levels. Both Li et al. (2020) and Feng et al. (2023) contributed substantially to the pooled effect, showing consistent directionality and narrow CIs, while Zhao et al. (2021) demonstrated a similar but non-significant trend. The analysis exhibited no heterogeneity (Q = 0.47, df = 2, *p* = 0.79; I² = 0%), indicating agreement among studies while still requiring cautious interpretation because of the small number of included studies. Clinically, these results underscore the potential pathogenic role of elevated AA, a pro-inflammatory omega-6 metabolite, in retinal microvascular dysfunction. AA serves as a precursor for eicosanoids, including prostaglandins and leukotrienes, which may contribute to inflammatory and vascular pathways relevant to the diabetic retina. Thus, the balance between omega-6 and omega-3 pathways and the role of AA accumulation warrant further evaluation in relation to retinal inflammation and DR onset in diabetic patients.

#### Predictive performance of omega-6 lipid models for DR

**Fig. 7:**
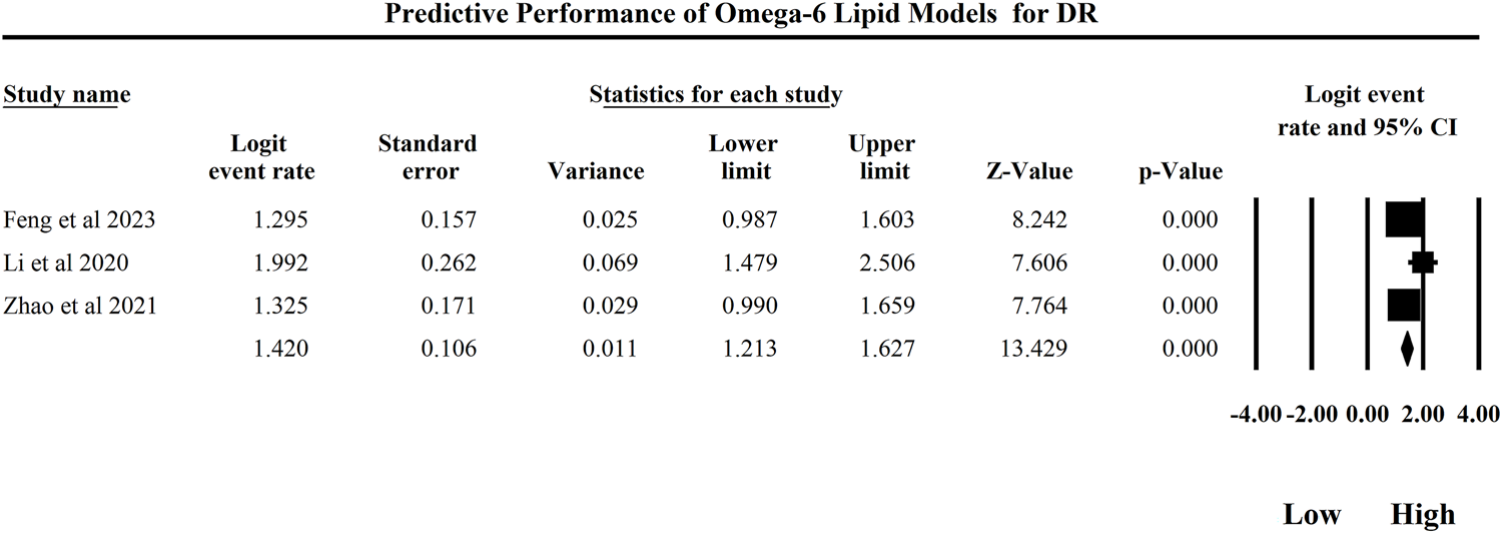
Forest plot of logit-transformed event rates derived from predictive-model studies evaluating omega-6-related lipid signatures for diabetic retinopathy (DR). The plotted estimates are logit-transformed event rates and should not be interpreted as raw area under the receiver operating characteristic curve values.

The exploratory synthesis evaluating predictive-model performance based on omega-6 fatty acid profiles demonstrated promising discriminative ability for predicting DR, as depicted in Fig. 7. Because Fig. 7 presents logit-transformed estimates rather than raw AUC values, these results should not be interpreted as an AUC greater than 1. Individual studies reported AUC values in the approximate range of 0.79–0.92, suggesting promising but not yet clinically validated discriminative performance. Models incorporating omega-6 lipid biomarkers, such as LA, AA, and γ-linolenic acid (GLA), showed promising discrimination in differentiating DR from non-DR cases in individual studies. Among the included studies, Feng et al. (2023) contributed the highest weight due to its comprehensive multivariate modeling and lowest standard error, followed by Li et al. (2020) and Zhao et al. (2021), both of which demonstrated consistent diagnostic trends. Heterogeneity was moderate (Q = 5.72, df = 2, *p* = 0.057; I² = 65%), indicating variability across studies and supporting cautious interpretation of the exploratory synthesis. Clinically, these findings suggest that omega-6-derived lipid signatures may serve as candidate biochemical predictors of DR, pending external validation. Integrating such metabolic biomarkers into predictive models may facilitate earlier detection of retinal microvascular damage in diabetes after further validation.

#### Adverse events associated with omega-6 fatty acid exposure in DR

**Fig. 8:**
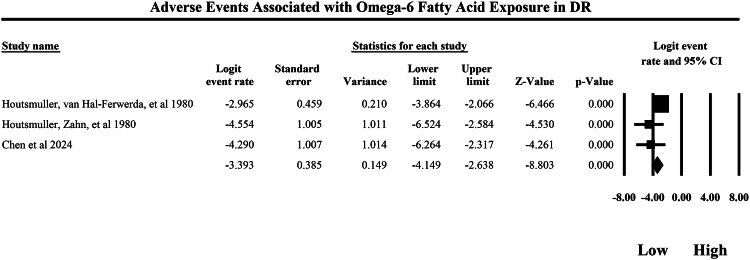
Forest plot of logit-transformed event rates for reported adverse or non-retinal clinical outcomes associated with omega-6 fatty acid exposure in diabetic retinopathy (DR). The plotted estimates are logit-transformed event rates for reported adverse or non-retinal clinical outcomes.

The exploratory synthesis of three studies evaluating the relationship between omega-6 fatty acid exposure and reported adverse or non-retinal clinical outcomes among DR patients is shown in Fig. 8. The pooled fixed-effects estimate yielded a logit event rate of −3.39 (95% CI, −4.15 to −2.64; *p* < 0.001), but this estimate should be interpreted cautiously because the included studies differed in exposure type, outcome definition, follow-up duration, and adverse-event ascertainment. In logistic terms, this corresponds to an event probability of approximately 3.2%; however, the available evidence remains insufficient to conclude that omega-6 supplementation or higher circulating levels are definitively safe across diabetic populations. Among the included studies, Houtsmuller & van Hal-Ferwerda (1980) and Chen et al. (2024) contributed the highest statistical weights due to precise estimates and narrow CIs. Heterogeneity across studies was low (Q = 2.99, df = 2, *p* = 0.22; I² = 33.3%), although the small number of studies limits the certainty of this exploratory estimate. Clinically, these findings did not identify a consistent signal suggesting that omega-6 fatty acid-based exposure exacerbates diabetic complications, but prospective safety evaluation remains necessary. These findings support further prospective evaluation of omega-6 fatty acids as adjunct nutritional strategies for microvascular protection in DR management.

#### Publication bias

Publication bias assessments were exploratory because each meta-analytic outcome included only a small number of studies. Begg and Mazumdar’s rank correlation test, Egger’s regression test, Duval and Tweedie’s trim-and-fill method, and funnel plot inspection were used descriptively. For DR occurrence or presence, Begg’s test yielded Kendall’s tau = –0.40 (*p* = 0.26) and Egger’s intercept = –1.26 (*p* = 0.07); no missing studies were identified by trim-and-fill, but these findings should be interpreted cautiously because the analysis was underpowered. In the LA subgroup, both Begg’s (τ = –0.17, *p* = 0.73) and Egger’s (*p* = 0.41) tests were nonsignificant, but this did not exclude publication bias. Similarly, the arachidonic acid analysis showed no clear asymmetry signal (τ = 0.00, *p* = 1.00; Egger’s intercept = 0.68, *p* = 0.48), with trim-and-fill imputing one potentially missing study that did not materially alter pooled estimates. For reported adverse or non-retinal clinical outcomes, Begg’s test (τ = 0.00, *p* = 1.00) and Egger’s test (*p* = 0.07) showed no clear asymmetry signal, and trim-and-fill identified no missing studies. Finally, for predictive model accuracy, Begg’s correlation (τ = 0.67, *p* = 0.30) and Egger’s intercept (*p* = 0.07) indicated mild asymmetry but not at a statistically significant level. Across all outcomes, the fail-safe N values ranged from 12 to 861; these results should be interpreted as exploratory. No clear evidence of publication bias was observed, but these analyses were underpowered and should not be interpreted as excluding publication bias.

## Discussion

This systematic review and meta-analysis suggests that omega-6 fatty acid-related measures are associated with diabetic retinopathy in a metabolite-specific manner. Higher circulating or dietary omega-6 levels, particularly linoleic acid (LA), were associated with lower odds of diabetic retinopathy (DR), as shown in Figs. 4 and 5, whereas arachidonic acid (AA) and selected downstream metabolites were associated with higher odds, as shown in Fig. 6. These findings support the biological plausibility that omega-6 pathways may influence retinal microvascular vulnerability, but they should be interpreted cautiously because the included studies differed in design, exposure measurement, outcome ascertainment, and adjustment for confounding, as summarized in Table 3. Predictive models incorporating omega-6 lipid biomarkers reported promising discrimination, but their clinical use requires further validation because Fig. 7 presents logit-transformed predictive-model estimates rather than raw area under the receiver operating characteristic curve values. Available dietary intervention studies did not identify a clear increase in reported adverse or non-retinal clinical outcomes with linoleic acid-rich diets, but the safety evidence was limited and was not uniformly collected across studies, as shown in Fig. 8.

Clinically, these findings suggest that dietary optimization of omega-6 intake, particularly through LA-rich sources such as vegetable oils and nuts, may be associated with preservation of retinal microvascular integrity and delayed DR onset. However, excessive conversion to pro-inflammatory AA derivatives should be interpreted in the context of dietary balance with omega-3 fatty acids. Collectively, this synthesis supports omega-6 fatty acid-related measures as clinically relevant and nutritionally modifiable candidate biomarkers that may complement holistic DR prevention strategies alongside glycemic management. When considered together with prior evidence suggesting a protective association between omega-3 fatty acid intake and DR, our findings support a broader paradigm in which balanced PUFA profiles—rather than single nutrient targets—shape retinal microvascular vulnerability in diabetes. The integration of omega-6 biomarker assessment into routine diabetic monitoring holds promise for personalized, preventive ophthalmic care.

Higher levels of specific omega-6 fatty acids, particularly LA and DGLA, were generally associated with reduced incidence and progression of DR, whereas elevated AA metabolites, such as 12-HETE, were linked to increased disease risk [28, 36, 37]. This duality reflects the complex metabolic roles of omega-6 fatty acids, associated with both pro-inflammatory and anti-inflammatory derivatives [38].

Interestingly, Zhao et al. (2021) reported an unexpected association between higher total ω-3 PUFA levels and increased DR risk (OR: 5.34, 95% CI: 2.48–11.52), which contrasts with the generally protective role attributed to ω-3 fatty acids in vascular health. This discrepancy may be explained by methodological differences, as Zhao et al. measured total ω-3 PUFAs without distinguishing specific subtypes such as EPA or DHA, which have distinct metabolic and anti-inflammatory properties. Furthermore, diabetic individuals with advanced disease often exhibit altered lipid metabolism, where elevated circulating ω-3 levels may reflect compensatory metabolic dysregulation rather than beneficial intake. Another plausible explanation is dietary imbalance—where higher ω-3 intake without adequate ω-6 proportion could disrupt the physiological ω-6/ω-3 equilibrium, influencing retinal microvascular function. Therefore, the findings of Zhao et al. highlight the importance of evaluating both total and relative fatty acid compositions when assessing the risk of DR, and they underscore the need for future studies to explore the mechanistic pathways underlying divergent effects of ω-3 and ω-6 PUFAs.

Dietary intervention studies showed the protective role of omega-6 fatty acids. Long-term consumption of an LA-enriched diet significantly reduced DR progression, particularly among individuals with poor glycemic control [31]. This highlights the importance of dietary modulation of fatty acids in affecting retinal microvascular outcomes. Conversely, studies measuring circulating 12-HETE consistently identified it as a predictor of DR onset [28]. This may be associated with its pro-inflammatory action on endothelial cells and promotion of leukocyte adhesion [39]. These suggest that the impact of omega-6 fatty acids may depend on the balance between protective and harmful metabolites.

Genetic analyses further support a protective association, with Mendelian randomization studies demonstrating reduced DR risk in individuals with genetically higher circulating omega-6 levels, particularly in type 2 diabetes [35]. The meta-analysis of predictive models confirmed that omega-6-related biomarkers showed strong diagnostic performance, with pooled AUC values exceeding 0.80 and sensitivity and specificity above 70% [28, 37]. These findings are in line with our previous work applying advanced analytic approaches—including network meta-analysis and multimodal prediction models—to other retinal diseases, which collectively support the feasibility of integrating biochemical, imaging, and clinical data for individualized risk stratification [40]. Incorporating omega-6 lipid signatures into such frameworks could further refine early detection strategies for DR.

On the other hand, omega-6 supplementation, particularly in the form of LA-rich diets, was associated with minimal adverse events [30, 31]. Similarly, higher LA intake offered cardiovascular protection, consistent with prior literature showing inverse associations between LA consumption and cardiovascular mortality.

### Study strengths and limitations

This comprehensive synthesis evaluated the role of omega-6 fatty acids in DR, integrating data from diverse geographical regions, including Asia, Europe, and the Middle East. This breadth enhances generalizability to different populations with varying dietary patterns. In addition, the systematic inclusion of both dietary intervention and biomarker-based studies provided complementary insights into the mechanistic and clinical aspects of omega-6 fatty acids. Moreover, predictive performance, as measured by AUC, sensitivity, and specificity, quantitatively demonstrated the diagnostic potential of omega-6 fatty acids.

However, heterogeneity in the methods of fatty acid measurement (e.g., LC-MS/MS, GC-MS, UPLC-MS) potentially introduces assay-related variability. Furthermore, DR diagnosis and staging were not standardized across studies, with some employing fluorescein angiography while others relied on fundus photography or clinical grading, which may bias outcome classification. Moreover, few studies adjusted for confounders such as glycemic control, duration of diabetes, or use of lipid-lowering medications, which limits causal attribution.

### Study implications and future research directions

The analysis emphasizes the importance of monitoring circulating omega-6 fatty acid profiles, which may enhance risk stratification for DR and provide a basis for biomarker-driven early detection strategies. Dietary guidance emphasizing a balanced intake of omega-6 fatty acids, particularly LA, could complement glycemic control in reducing DR progression. This is particularly relevant in light of accumulating evidence from our previous systematic reviews demonstrating that systemic agents such as fibrates, statins, and other metabolic modulators can modify DR risk and progression [41, 42]. Additionally, it is crucial to consider metabolites such as 12-HETE, which are associated with DR pathology.

Nevertheless, future research should explore the roles of individual omega-6 metabolites, clarify dose-response relationships, and evaluate interactions with omega-3 fatty acids and other dietary components. In addition, studies should investigate the influence of targeted dietary or pharmacological modulation of omega-6 pathways on DR outcomes. Integrating lipidomics with genomic and clinical data could help establish precision nutrition and personalized interventions for diabetic patients at risk of retinopathy.

## Conclusions

This systematic review and meta-analysis provides quantitative evidence that omega-6 fatty acid-related measures are associated with DR in a metabolite-specific manner. Higher total omega-6 and LA measures were associated with lower odds of DR, whereas higher AA levels were associated with higher odds. These findings suggest that the balance between protective omega-6 species and pro-inflammatory downstream metabolites may be relevant to retinal microvascular health in diabetes. Predictive models incorporating omega-6 lipid biomarkers showed promising discrimination in individual studies, but these findings require external validation before clinical implementation. Available safety data did not identify a consistent adverse signal, but they remain sparse and heterogeneous. Overall, omega-6 fatty acid profiling may represent a promising adjunctive biomarker strategy for DR risk stratification, but further prospective studies using standardized exposure measurement, retinal outcome definitions, and confounder adjustment are required.

## Supplementary information

Supplementary information

## Data Availability

All data generated or analyzed during this study are included in this published article and its supplementary information files. No new datasets were generated or used.
