# Supplementary material for: Association between omega-6 fatty acids and diabetic retinopathy risk: a systematic review and meta-analysis
Source: Nutr Diabetes. 2026 Jun 11;16:26. doi: 10.1038/s41387-026-00417-x (PMC13442842; doi:10.1038/s41387-026-00417-x)
Supplement: Supplementary file 1 [file 41387_2026_417_MOESM1_ESM.docx]

Supplementary Figures

Supplementary Figure 1: Funnel plot of the incidence of diabetic retinopathy (DR).

Supplementary Figure 2: Funnel plot of linoleic acid.

Supplementary Figure 3: Funnel plot of arachidonic acid.

Supplementary Figure 4: Funnel plot of predictive models.

Supplementary Figure 5: Funnel plot of adverse events.
